# Characterization of BK polyomavirus-associated nephropathy (BKPyVAN) in kidney transplant recipients using transcriptomic analysis: a comprehensive scoping review

**DOI:** 10.1093/femspd/ftaf006

**Published:** 2025-07-24

**Authors:** Lachlan A Davidson, Natalie M Niessen, Matthew Rowlandson, Thida M Myint, Paul R Trevillian, Adrian D Hibberd, Munish K Heer, Jay C Horvat, Katherine J Baines

**Affiliations:** School of Biomedical Sciences and Pharmacy, College of Health, Medicine and Wellbeing, The University of Newcastle, Callaghan, NSW 2308, Australia; Immune Health Research Programme, Hunter Medical Research Institute, New Lambton Heights, NSW 2305, Australia; Hunter Transplant Research Foundation, John Hunter Hospital, New Lambton Heights, NSW 2305, Australia; School of Biomedical Sciences and Pharmacy, College of Health, Medicine and Wellbeing, The University of Newcastle, Callaghan, NSW 2308, Australia; Immune Health Research Programme, Hunter Medical Research Institute, New Lambton Heights, NSW 2305, Australia; Hunter Transplant Research Foundation, John Hunter Hospital, New Lambton Heights, NSW 2305, Australia; Newcastle Renal Transplant Unit, John Hunter Hospital, New Lambton Heights, NSW 2305, Australia; Newcastle Renal Transplant Unit, John Hunter Hospital, New Lambton Heights, NSW 2305, Australia; Hunter Transplant Research Foundation, John Hunter Hospital, New Lambton Heights, NSW 2305, Australia; Newcastle Renal Transplant Unit, John Hunter Hospital, New Lambton Heights, NSW 2305, Australia; Hunter Transplant Research Foundation, John Hunter Hospital, New Lambton Heights, NSW 2305, Australia; Newcastle Renal Transplant Unit, John Hunter Hospital, New Lambton Heights, NSW 2305, Australia; Hunter Transplant Research Foundation, John Hunter Hospital, New Lambton Heights, NSW 2305, Australia; Newcastle Renal Transplant Unit, John Hunter Hospital, New Lambton Heights, NSW 2305, Australia; School of Biomedical Sciences and Pharmacy, College of Health, Medicine and Wellbeing, The University of Newcastle, Callaghan, NSW 2308, Australia; Immune Health Research Programme, Hunter Medical Research Institute, New Lambton Heights, NSW 2305, Australia; School of Biomedical Sciences and Pharmacy, College of Health, Medicine and Wellbeing, The University of Newcastle, Callaghan, NSW 2308, Australia; Immune Health Research Programme, Hunter Medical Research Institute, New Lambton Heights, NSW 2305, Australia; Hunter Transplant Research Foundation, John Hunter Hospital, New Lambton Heights, NSW 2305, Australia

**Keywords:** BK polyomavirus-associated nephropathy, BK polyomavirus, transcriptomics, infection, kidney transplant, scoping review

## Abstract

BK polyomavirus (BKPyV) infection reactivates with immunosuppressive therapies and can lead to the development of BKPyV-associated nephropathy (BKPyVAN) in kidney transplant recipients. This scoping review assesses the use of transcriptomics to profile BKPyVAN in kidney transplant recipients. The following search strategy was employed in Medline and Embase: ‘BK virus’ or ‘BK polyomavirus’ or ‘Polyomavirus’ AND ‘Kidney transplant’ or ‘Nephritis’ or ‘Nephropathy’ AND ‘Gene expression’ or ‘Transcriptomics’ or ‘mRNA’. The search identified 368 publications (264 EMBASE and 104 MEDLINE), and after removal of duplicates (92) and abstract/full-text screening, there were 11 eligible studies which included 7 original transcriptomic studies and 4 bioinformatic studies. There was consistent dysregulated expression of proinflammatory pathways (e.g. cytokines, chemokines, T- and B-cell-related pathways) in BKPyVAN compared with stable graft function. There was considerable overlap between the gene expression patterns identified in BKPyVAN and T-cell mediated rejection that require more exploration. This review highlights limitations including small sample sizes, lack of validation, and variation in technical platforms and study designs. Transcriptomics in BKPyVAN is currently underutilized and there is a genuine need for further research in larger cohorts to provide action in discovering novel therapeutic targets and discriminative gene expression signatures to guide individualized therapeutic strategies.

## Teaser

This study is the first to reveal that transcriptomic profiling of BKPyVAN and a definitive multicenter investigation is lacking, highlighting the genuine need for original research in this area.

Abbreviations BHOT
: BANFF human organ transplant
BKPyV
: BK polyomavirus
BKPyVAN
: BK polyomavirus-associated nephropathy
GO
: Gene ontology
GSEA
: Gene set enrichment analyses
IFN
: Interferon
IL
: Interleukin
KEGG
: Kyoto Encyclopedia of Genes and Genomes
KTR
: Kidney transplant recipient
mRNA
: Messenger ribonucleic acid
NCBI GEO
: National Centre for Biotechnology Information Gene Expression Omnibus
RT-PCR
: Real-time polymerase chain reaction
PD
: Programmed death
PRISMA-ScR
: Preferred Reporting Items for Systematic reviews and Meta-Analyses extension for Scoping reviews
QNAT
: Quantitative nucleic acid testing
SV40
: Simian vacuolating virus 40
TCMR
: T-cell mediated rejection
Th
: T helper
TNF
: Tumour necrosis factor

## Introduction

BK polyomavirus-associated nephropathy (BKPyVAN) pathology remains a significant contributor to allograft dysfunction in kidney transplant recipients (KTRs), representing a major risk for immunosuppressed individuals (Gately et al. 2023). Transplant teams worldwide have highlighted the difficulty of managing BKPyVAN in KTRs posttransplant, with significantly higher rates of graft dysfunction and loss, as well as rejection (Gately et al. 2023, Higdon et al. 2023, Kotton et al. 2024). Risk factors for biopsy-proven BKPyV-nephropathy include recipient older age, male sex, donor BKPyV-viruria, BKPyV-seropositive donor/-seronegative recipient, tacrolimus, acute rejection, and higher steroid exposure (Kotton et al. 2024). Understanding of BKPyVAN pathological mechanisms is far from complete, further emphasizing the difficulty of managing and balancing risk factors that patients may face. Limited evidence continues to pose a major drawback in the current understanding of BKPyVAN and several questions remain unanswered in this area. For example, it is not clear how BKPyV is reactivated in KTRs and whether the virus is of donor or recipient origin. In addition, it is unknown why some KTRs with BKPyV infection progress to immune-mediated pathology and tissue damage while others with similar management can recover from BKPyV infection with minimal graft inflammation. Several hypotheses for BKPyVAN have been proposed, including when the recipient receives a different strain of BKPyV from the donor, where the recipient immunity cannot control the reinfection with the new strain (Higdon et al. 2023). A higher degree of HLA mismatch between donor and recipient is associated with elevated risk of both BKPyVAN and acute rejection, suggesting that similar pathways are activated and that BKPyVAN may contribute to the development of acute rejection and *vice versa* (Higdon et al. 2023). There is currently no strong evidence for an antiviral treatment directly targeting BKPyV infection or BKPyVAN, therefore further research is needed to improve current management strategies and identify novel treatments that target BKPyV.

The diagnosis of BKPyVAN currently relies on anti-SV40 immunohistochemistry that identifies the expression of BKPyV large-T antigen in the nucleus of renal tubular epithelial cells, along with histological assessment of allograft biopsies using Banff histological classification [primarily acute markers, tubulitis (t), and interstitial inflammation (i)]. However, the role of blood and urine qualitative nucleic acid testing in BKPyVAN management remains unclear, as nephrologists are required to judiciously determine the magnitude of immunosuppression withdrawal based on BKPyV DNA quantitation in the blood.

The frequency and duration of BKPyV screening tests are highly variable between transplant centres, as the evidence is reliant upon observational data (Myint et al. 2022). These tests also may not be a true indicator of pathology in the kidney microenvironment, in some cases BKPyV viremia does not progress to BKPyVAN. Therefore, withdrawing immunosuppression from a patient diagnosed with BKPyV viremia (with no observed BKPyVAN pathology) is a counter-productive intervention strategy that may risk rejection (Chen et al. 2020). Current diagnostic methods struggle to distinguish between viral- and alloantigen-related inflammation leading to difficulty in clinical management. Hence, we propose that better diagnostic tools, such as gene expression signatures of both viral and immune-mediated markers in the kidney microenvironment, are required to facilitate the management of BKPyV infection and identify those at risk of BKPyVAN. Underlying factors that determine disease progression in BKPyV infection include a combination of innate and adaptive immune responses, type, duration and intensity of immunosuppression, the severity of BKPyV infection, genetic susceptibility and even coinfection (Myint et al. 2022). Overall, the inconclusive relationship between BKPyV infection and the immunological changes that occur in the allograft remains the major problem underlying the application of scientific research and clinical interpretation.

Management of BKPyVAN relies on the timely reduction of immunosuppression, which may trigger kidney transplant rejection. The complexity is compounded by the fact that T-cell mediated rejection (TCMR) exhibits similar clinical characteristics, histological features, and infiltrating cells to BKPyVAN but requires increased immunosuppression, making diagnosis and balancing therapies a challenging and ongoing issue for clinicians (Sigdel et al. 2016). Therefore, translating basic science research to the transplant clinic remains a monumental challenge. To bridge this knowledge gap, this manuscript performs a scoping review to evaluate the published literature on transcriptomic studies of BKPyVAN cohorts with a focus on gene expression signatures and cellular pathways.

## Methods

*Inclusion and exclusion criteria*: this scoping review was conducted based on the Preferred Reporting Items for Systematic reviews and Meta-Analyses extension for Scoping reviews (PRISMA-ScR) (Tricco et al. 2018). The purpose of this review was to assess the scope, nature and extent of the available literature on the use of transcriptomics to profile BKPyV-infected renal allografts in KTRs. Our search strategy included journal articles that were related to messenger ribonucleic acid (mRNA) gene expression profiling of BKPyV infection in renal allograft samples, leading to the identification of transcriptomic signatures, differentially expressed genes (DEG), and cellular pathways. This study will allow the inclusion of studies presenting original human clinical data (cohort, cross-sectional, randomized control trial, case-control, case-report, and case-series) and bioinformatic reviews of original BKPyVAN transcriptomic datasets. The articles must also be full-text and published in a peer-reviewed journal. Studies irrelevant to BKPyV infection and articles that did not discuss the transcriptomic evaluation of posttransplant renal allografts were excluded. Articles that did not contain gene expression data from human clinical cohorts (such as animal and *in vitro* models) were excluded. Manuscripts that did not provide mRNA gene expression profiling were excluded. Papers that were not published in a peer-reviewed journal or were not published in a full journal article format were excluded. Preprints, abstract-only texts, and languages other than English were also excluded.

*Search strategy*: bibliographic database searches were conducted on the 13th of December 2024, encompassing the OVID Embase and MEDLINE databases. The searches spanned up to the 13th of December 2024, with no restrictions on the starting year. The searches were conducted using the following keywords: ‘BK virus’ or ‘BK polyomavirus’ or ‘Polyomavirus’ AND ‘Kidney transplant’ or ‘Nephritis’ or ‘Nephropathy’ AND ‘Gene expression’ or ‘Transcriptomics’ or ‘mRNA’.

*Selection of sources of evidence*: the search results were imported and reviewed using Endnote 20 (Microsoft, Redmond, WA, USA). After duplications were removed, the inclusion and exclusion criteria guided the removal of irrelevant papers where two authors (LD and KB) independently checked the titles and abstracts for eligibility. Discussion between the two authors was conducted when there were discrepancies in the inclusion/exclusion of papers, with any final discrepancies resolved via a third reviewer (NN). Reasons for the exclusions were recorded.

*Data extraction and synthesis*: the following items were extracted from included papers: first author, date of submission/publication, type of study (original or bioinformatic), methodology of study (in the case of an original study), and number of patients studied in the original investigations.

## Results

### Literature search results

**Figure 1.**
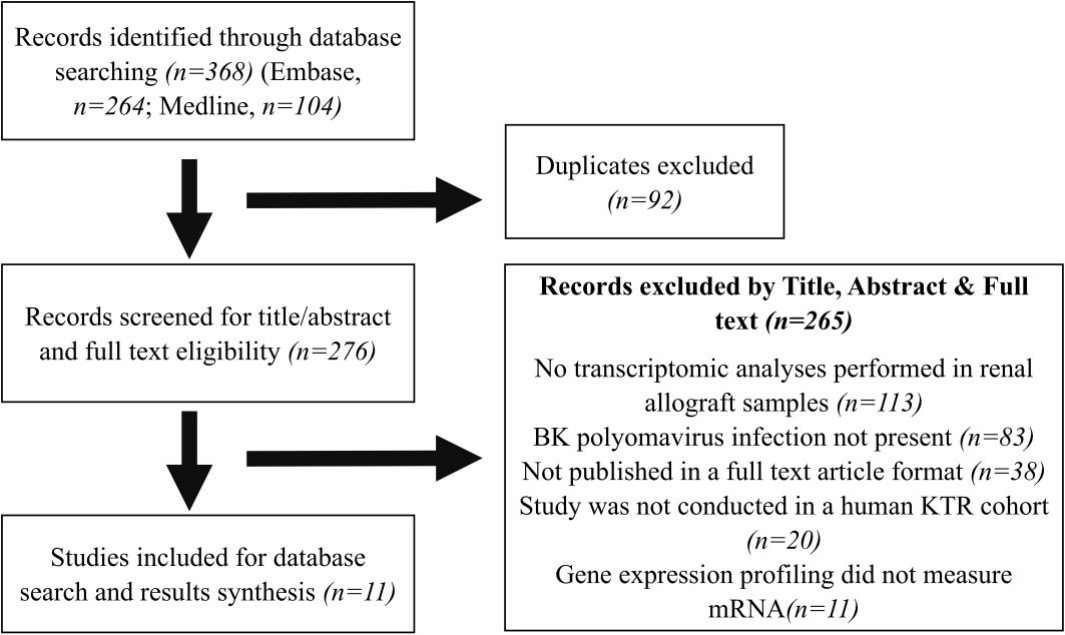
PRISMA flow chart, the process of study selection.

The results from the search strategy are outlined in Fig. 1. We retrieved 368 publications from our search (264 Embase and 104 Medline). After removing 92 duplicates, we screened the titles and abstracts of 276 publications for relevance and evaluated them for full-text eligibility, resulting in the exclusion of 265 publications. Of the 265 publications that were excluded, 113 did not report transcriptomic profiling in posttransplant renal allografts, 83 did not refer to BKPyV infection or BKPyVAN pathology, 38 were not published in a full text article format (abstract only), 20 did not report on human clinical cohorts (*in vitro* or animal models), 11 did not report on messenger RNA gene expression (primarily focused on microRNA), and all papers were published in the English language. Thus, data synthesis and extraction was conducted on the 11 publications that were included in this review (Fig. 1).

### Study cohort characteristics

The participant and study characteristics of the 11 studies included in this scoping review are shown in Table 1 (Mannon et al. 2005, Girmanova et al. 2012, Lubetzky et al. 2014, Sigdel et al. 2016, Jia et al. 2018, Adam et al. 2020, Liu et al. 2020, Halloran et al. 2021, Zhao et al. 2022, Sato et al. 2023, Yang et al. 2023). The first published paper dates to 2005, however the majority (*n* = 9) of the articles were published in the last decade (Lubetzky et al. 2014, Sigdel et al. 2016, Jia et al. 2018, Adam et al. 2020, Liu et al. 2020, Halloran et al. 2021, Zhao et al. 2022, Sato et al. 2023, Yang et al. 2023). Of the 11 publications, a total of 7 articles were original human clinical studies that investigated gene expression profiles of patients with BKPyVAN (Mannon et al. 2005, Girmanova et al. 2012, Lubetzky et al. 2014, Sigdel et al. 2016, Adam et al. 2020, Halloran et al. 2021, Yang et al. 2023). Mannon et al. (2005) undertook a polymerase chain reaction (PCR)-based array transcriptional evaluation of BKPyVAN, TCMR, and a stable functioning KTR cohort to identify how DEG may correlate to clinical histological characteristics, such as immune cell infiltration and fibrotic activity. Girmanova et al. (2012) conducted PCR-based array profiling of three patient groups (BKPyVAN, BKPyV viruria, and a stable functioning KTR group), where they also performed a gene ontology (GO) analysis of their differentially expressed mRNA candidates. Lubetzky et al. (2014) compared BKPyVAN, BKPyV viremia, and stable functioning KTR group with a whole genome microarray and performed a pathway analysis using GO. Sigdel et al. (2016) measured BKPyVAN, TCMR, stable functioning, and interstitial fibrosis/tubular atrophy KTR cohorts using a whole genome microarray to identify BKPyVAN-specific genes. Adam et al. (2020) designed a discovery cohort that contained a native kidney BKPyVAN (*n* = 5) and pure TCMR (*n* = 10) group and a validation cohort measuring allograft BKPyVAN (*n* = 60) and TCMR (*n* = 10) groups with a Nanostring digital-based array, to discover and validate a viral and host transcriptomic signature to distinguish the two pathologies. Halloran et al. (2021) conducted a whole genome microarray with 50 BKPyVAN-positive and 52 BKPyVAN-negative renal allograft biopsies, where a single BK viral transcript (*VP2*) conducted via real time-polymerase chain reaction (RT-PCR) was correlated to gene expression of host markers and clinical characteristics. Yang et al. (2023) provided a single-cell transcriptomic analyses of BKPyVAN posttransplant kidneys (*n* = 3) and a noninfected control group (*n* = 10) to identify DEG that were specific to BKPyV infection of renal proximal tubule cells in KTRs.

**Table 1.** The summary of studies identified by our search strategy from each journal article are reported.

References	Platform	Sample groups	Dataset
Human clinical studies			
Mannon et al. (2005)	Taqman Low Density Array (qPCR panel)	Stable: *n* = 15 BKPyVAN: *n* = 10 Rejection: *n* = 17	N/A
Girmanova et al. (2012)	Taqman Low Density Array (qPCR panel)	Stable: *n* = 11 BKPyVAN: *n* = 10 BKPyV viruria: *n* = 9	N/A
Lubetzky et al. (2014)	Affymetrix 1.0 (whole genome microarray)	Stable: *n* = 11 BKPyVAN: *n* = 3 BKPyV viremia: *n* = 3	GSE47199
Sigdel et al. (2016)	Affymetrix 2.0 (whole genome microarray)	Stable: *n* = 73 BKPyVAN: *n* = 10 TCMR: *n* = 26 Interstitial fibrosis/tubular atrophy (IFTA): *n* = 59	GSE72925
Adam et al. (2020)	Nanostring PanCancer panel (digital gene expression panel)	Discovery cohort Native BKPyVAN: *n* = 5 Pure TCMR: *n* = 10 Validation cohort BKPyVAN: *n* = 60 Pure TCMR: *n* = 10	N/A
Halloran et al. (2021)	Affymetrix microarray (whole genome-based array)	BKPyVAN-positive: *n* = 50 BKPyVAN-negative: *n* = 52	N/A
Yang et al. (2023)	BD rhapsody single-cell whole transcriptome assay	BKPyVAN-positive (*n* = 3) BKPyVAN-negative (*n* = 10)	HRA002472, GSE195790, GSE131685, GSE171458
Bioinformatic studies			
Jia et al. (2018)	Affymetrix 2.0 (whole genome-based array)	Stable: *n* = 30 BKPyVAN: *n* = 15	GSE75693
Liu et al. (2020)	GSE75693 and GSE47199, details above		
Zhao et al. (2022)	GSE75693, GSE47199 and GSE72925, details above		
Sato et al. (2023)	GSE75693, GSE47199 and GSE72925, details above		

Of the 11 publications, 4 were bioinformatic studies that analysed the NCBI GEO datasets (GSE72925, GSE75693, and GSE47199) containing BKPyVAN patient cohorts (Jia et al. 2018, Liu et al. 2020, Zhao et al. 2022, Sato et al. 2023). Jia et al. (2018) conducted a bioinformatic analysis of the GEO dataset (GSE75693) to identify DEG, which was originally conducted on a whole genome microarray with BKPyVAN (*n* = 15) and stable functioning allograft (*n* = 30) patients. The study identified key hub genes for BKPyVAN, along with pathway analyses using GO and Kyoto Encyclopedia of Genes and Genomes (KEGG) (Jia et al. 2018). Liu et al. (2020) collected BKPyVAN microarray data (GSE47199 and GSE75693), where the results of gene set enrichment analysis (GSEA) and single-sample GSEA showed that immune cell-, cytokine-, chemokine-, and inflammation-related pathways were significantly activated in BKPyVAN compared to stable allografts, whereas metabolism- and renal development-related pathways were significantly downregulated. Zhao et al. (2022) studied the microarray datasets GSE75693, GSE47199, and GSE72925 with a specific focus on endoplasmic reticulum stress during BKPyVAN pathology. Endoplasmic reticulum-related hub genes and endoplasmic reticulum pathways were identified with GO and KEGG analyses. Sato et al. (2023) also examined the microarray datasets GSE75693, GSE47199, and GSE72925 in addition to GSE120495 with a focus on specific transcriptomic signatures and pathogenic pathways that are shared between BKPyVAN and renal cell carcinoma. Most studies that analysed original data had a BKPyVAN sample size ≥10 (*n* = 6) (Mannon et al. 2005, Girmanova et al. 2012, Sigdel et al. 2016, Jia et al. 2018, Adam et al. 2020, Halloran et al. 2021), with the number of BKPyVAN allograft samples in a cohort ranging from 3 to 60. There was also a range of different transcriptomic technologies employed including whole genome gene expression microarrays (*n* = 4) (Lubetzky et al. 2014, Sigdel et al. 2016, Jia et al. 2018, Halloran et al. 2021), PCR based arrays (*n* = 2) (Mannon et al. 2005, Girmanova et al. 2012) and a digital gene expression panel (*n* = 1) (Adam et al. 2020) and single-cell arrays (*n* = 1) (Yang et al. 2023).

The statistical methods used to identify DEG within each investigation varied considerably. In the earlier investigations, there were two studies (Mannon et al. 2005, Sigdel et al. 2016) that used parametric testing consisting of one-way analysis of variance and Bayesian *t*-test, three studies (Girmanova et al. 2012, Liu et al. 2020, Yang et al. 2023) conducted nonparametric testing of which all were Mann–Whitney U tests, and one study (Adam et al. 2020) provided a linear regression to identify differential gene expression. Whereas in the most recent investigations, four studies (Jia et al. 2018, Liu et al. 2020, Halloran et al. 2021, Zhao et al. 2022) employed the ‘limma’ R package, where linear models were used to assess differential gene expression and one study (Sato et al. 2023) provided a weighted gene coexpression network analysis (WGCNA) in R library.

### Altered genes in BKPyVAN

Host-related gene expression biomarkers that were associated with BKPyVAN were extracted from the written text, tables, figures, and supplementary material of the 11 studies, where the identified gene symbols and study of origin were reported (Table 2). In this review, we specifically highlighted genes associated with immunological alterations in BKPyVAN pathology. The BKPyVAN and stable comparisons were undertaken in five studies (Mannon et al. 2005, Girmanova et al. 2012, Lubetzky et al. 2014, Zhao et al. 2022, Yang et al. 2023), while two studies (Sigdel et al. 2016, Halloran et al. 2021) completed a comparison between BKPyVAN and all other experimental groups (acute rejection/stable) to reveal genes that were BKPyVAN-specific. It was shown that six studies had chemokine-related gene alterations (Mannon et al. 2005, Girmanova et al. 2012, Lubetzky et al. 2014, Sigdel et al. 2016, Jia et al. 2018, Halloran et al. 2021), T-cell signalling genes were observed in four studies (Mannon et al. 2005, Girmanova et al. 2012, Lubetzky et al. 2014, Sigdel et al. 2016), proinflammatory molecules and cell-related activity genes in four studies (Mannon et al. 2005, Girmanova et al. 2012, Sigdel et al. 2016, Jia et al. 2018), and cytotoxicity-related genes in four studies (Mannon et al. 2005, Girmanova et al. 2012, Sigdel et al. 2016, Jia et al. 2018). Other DEG were also shown to be upregulated in BKPyVAN, these included complement-related genes in three studies (Girmanova et al. 2012, Sigdel et al. 2016, Jia et al. 2018), HLA class II-related genes in two studies (Girmanova et al. 2012, Lubetzky et al. 2014), immunoglobulin (*IGHM*) in two studies (Sigdel et al. 2016, Halloran et al. 2021), and macrophage signalling genes in two studies (Girmanova et al. 2012, Sigdel et al. 2016).

**Table 2.** The summary of DEG (BKPyVAN versus Stable) and the studies where they were reported.

Citation	Differential gene expression reported (BKPyVAN versus Stable KTR)
Mannon et al. (2005)	*Upregulated* ***↑*** *Chemokines—RANTES* *T-cell signalling/activation—CD3, CD154, CD80, CD86* *Proinflammatory molecules and cell-related activity—TNFα, TNFβ, FN1, VIM, CTGF, S100A4, T-bet, TGFβ, MMP2, MMP9* *Cell death and Cytotoxicity—Granzyme B, LTb, FasL, Bax, Granulysin*
Girmanova et al. (2012)	*Upregulated* ***↑*** *Chemokines—CCL2, CCL3, CCL5, CCR2, CCR7, CXCL10, CXCR3* *T-cell signalling—CD4, CD28, CD86* *Macrophages and Monocytes signalling—CD68* *HLA class II—HLADRA, HLADRB1* *Proinflammatory molecules and cell-related activity—IFNG, IL12B, IL1B, IL2RA, IL6, IL8, LTA, TNF, CSF, PTPRC, TBX21, TNFRSF18, ICAM1, TGFB* *Cell death and Cytotoxicity—PRF1, EDN1, FASL, GNLY* B-cell signalling—CD19 Complement—*C3*
Lubetzky et al. (2014)	*Upregulated* ***↑*** *Chemokine—CCR7* *HLA class II—HLA-DOB* *T-cell signalling—CD40 L (CD154)* *Proinflammatory molecules—CD33* *Toll-like receptor—TLR10* *Inhibitory signalling—BTLA, SLAMF1, KLRB1*
Sigdel et al. (2016) Note* BKPyVAN versus combined rejection/stable cohort	*Upregulated* ***↑*** *Complement—C1QA, C1QB, C1QC, C1S, C2, C3, C3AR1* *Chemokines—CCL18, CCL19, CCL2, CCL3, CCL5, CXCL10, CXCL13, CXCL6, CXCL9* *T-cell signalling—CD2, CD3D, CD48, CD52, CD69* *Macrophage signalling—CD14, CD163* *Cell death and Cytotoxicity—GZMA, GZMB, GZMK, PRF1* *Proinflammatory molecules and cell-related activity—CASP1, IL32, STAT1, ADA, AGR2, AGRN, AIF1, ALS2CR8, AMACR, ANKRD19, AP2S1, APOBEC3G, APOC1, ARHGAP15, ASL, ATN1, AURKAIP1, AVIL, BCL2A1, BIN2, BIRC3, BST2, BTN3A2, C11orf2, C11orf83, C1orf162, C4orf48, C9orf16, CARD16, CCDC109B, CCDC93, CD53, CDH6, CDKN1A, CFD, CHEK2, CHIC1, CKS2, CLEC2B, CNN1, CORO1A, CSPG5, CST7, CSTA, CTSS, CYTIP, DPP3, EDF1, EEF1D, EIF3G, EVI2A, F13A1, FAM101B, FAM105B, FAM26F, FBXL15, FCER1G, FCGR1A, FCGR1B, FGFR1, FKBP11, GABBR1, GBP1, GBP2, GBP5, GMFG, GPR183, GPX4, GYPC, HBA2, HCLS1, HCP5, HCST, HOPX, HPR, HSPD1, HTR6, ICAM2, IDO1, IFI27, IFITM1, IGKJ5, IRF1, ISG20, ITGAL, ITGB2, KLC4, KLRB1, LAPTM5, LARP4, LCP1, LENEP, LMOD1, LOC348262, LOC643187, LOC91316, LSM7, LSP1, LST1, LTB, LTF, LYG1, MIR142, MIRHG2, MMP7, MRPL54, MRPL55, MS4A4A, MS4A6A, N4BP1, NCAPH2, NCKAP1L, NDUFA11, NDUFA3, NDUFS6, NKAIN4, NKG7, NLRC5, NNMT, NOSIP, PHPT1, PLAC8, PLTP, POLR2I, PSMB10, PSMB8, PSMB9, PTGDS, PTPRC, PTPRCAP, PTTG1, RAC2, RARRES1, RARRES3, RASSF5, RBMS3, REG1A, RGS2, RNASEH2C, RPS15, S100A1, S100A8, SAA1, SAMD10, SCO2, SEC61A1, SF385, SLA, SLAMF7, SLAMF8, SLC1A3, SNRPB, SOD2, SPA17, STAB1, STK17A, TAF10, TAP1, TIMM13, TIMP1, TKT, TMC8, TMEM54, TNFRSF25, TNFSF13B, TNMD, TRAF3IP3, TRAPPC5, TRBC1, TRMT61A, TYROBP, UBE2D4, UBE2L6, WARS, WFDC2, WNK2, XCL1, ZNF511* *Immunoglobulin—IGHM*
Halloran et al. (2021) Note* BKPyVAN versus combined rejection/stable cohort	*Upregulated* ***↑*** *Chemokine—CXCL13* *B-cell signalling—CD19* *Immunoglobulins— IGHM* *Injury—RRM2* *BK viral gene—VP2* *Proinflammatory molecules and cell-related activity—PIF1, IGLL5, CDC6, IGFL2, ASF1B, KIAA0101, HSH2D, ADAMDEC1, FEN1, RA51AP1, CCNE2, LMNB1, ESCO2, UBE2J1, FANCI, GINS4, IGK, FAM111B, NCAPG, ZBP1, SAA1, CEP128, DAPP1, CDCA5, DTL, LMAN1*
Jia et al. (2018)	*Upregulated* ***↑*** *Chemokines—CCL5, CXCL9, CXCL10* *Proinflammatory molecules and cell-related activity—CASP1, STAT1, SMAD4, CDH1, CALM, TYROBP, PTPRC, HCK, IRF7, GBP5, PLEK, CD163, GBP2, GBP1, BIRC5* *Complement—C1QB, C1QA* *Cell death and cytotoxicity—GZMB* *Downregulated* ***↓*** *Tissue repair—EGF*
Yang et al. (2023) Note* BKPyV infection specific to renal proximal tubule epithelial cells	*Upregulated* ***↑*** *Cell proliferation—TMPO, MKI67*

BKPyVAN pathology was characterized by a broad spectrum of host-transcriptomic markers, mainly consisting of immune-related transcripts that are altered in both BKPyVAN and TCMR pathologies. Each study highlights the importance of various immune response pathways and the important role they play in BKPyVAN. However, many of the transcriptomic biomarkers identified in past BKPyVAN studies are also upregulated in rejection cohorts, which suggests that the changes observed are not BKPyVAN-specific and may be the result of concomitant TCMR, or cellular pathways are altered similarly in both pathological states. The report of Adam et al. (2020) suggested that an identified 7-immune-gene signature could not accurately discriminate between BKPyVAN and TCMR and that a BKPyVAN 5-viral gene expression could more accurately discriminate between BKPyVAN and pure TCMR. Differential gene expression observed in BKPyVAN compared with acute rejection was limited to four studies (Mannon et al. 2005, Sigdel et al. 2016, Adam et al. 2020, Halloran et al. 2021) [two studies directly compared a BKPyVAN and TCMR group (Table 3), while two other studies compared BKPyVAN to a combined stable/rejection group], and thus further investigations are needed to elucidate the relationship between BKPyVAN and rejection. Adam et al. (2020) proposed a BK polyomavirus 5-gene signature (*LTAg, VP1, VP2, Agnoprotein*, and *VP3*) that was able to diagnose BKPyVAN highly accurately. Halloran et al. (2021) also proposed a single viral transcript (*VP2*) as a highly selective predictive marker for BKPyVAN, from the comparison of BKPyVAN and a combined rejection/stable group. In addition, the expression of BKPyV *VP2* mRNA was correlated with *RRM2* (injury-associated gene), immunoglobulin mRNA (atrophy-fibrosis), and *CXCL13* (macrophage activation).

**Table 3.** The summary of DEG (BKPyVAN versus TCMR) and the studies where they were reported.

Citation	Differential gene expression reported (BKPyVAN versus acute rejection/TCMR)
Mannon et al. (2005)	Upregulated **↑** *T-cell signalling—CD8* *Chemokine- CXCR3* *Proinflammatory molecules and cell-related activity—IFNγ, COLIVA5, COLIA1, FN1, S100A4, αSMA, MMP2, MMP9, BMP7* *Cell death and Cytotoxicity—Perforin* *HLA class II—HLA-DR* *Growth Factor—TGFβ*
Adam et al. (2020)	Upregulated **↑** Chemokine—CXCL6 Immunoglobulins—FCGR2B *Proinflammatory molecules and cell-related activity—*MAP3K5, MEF2C Dendritic cells—CD1C BK Viral proteins—VP1, VP2, VP3, Agnoprotein, LTAg Downregulated **↓** *Proinflammatory molecules and cell-related activity—ITGA6, VEGFA*

### Altered pathways in BKPyVAN

**Figure 2.**
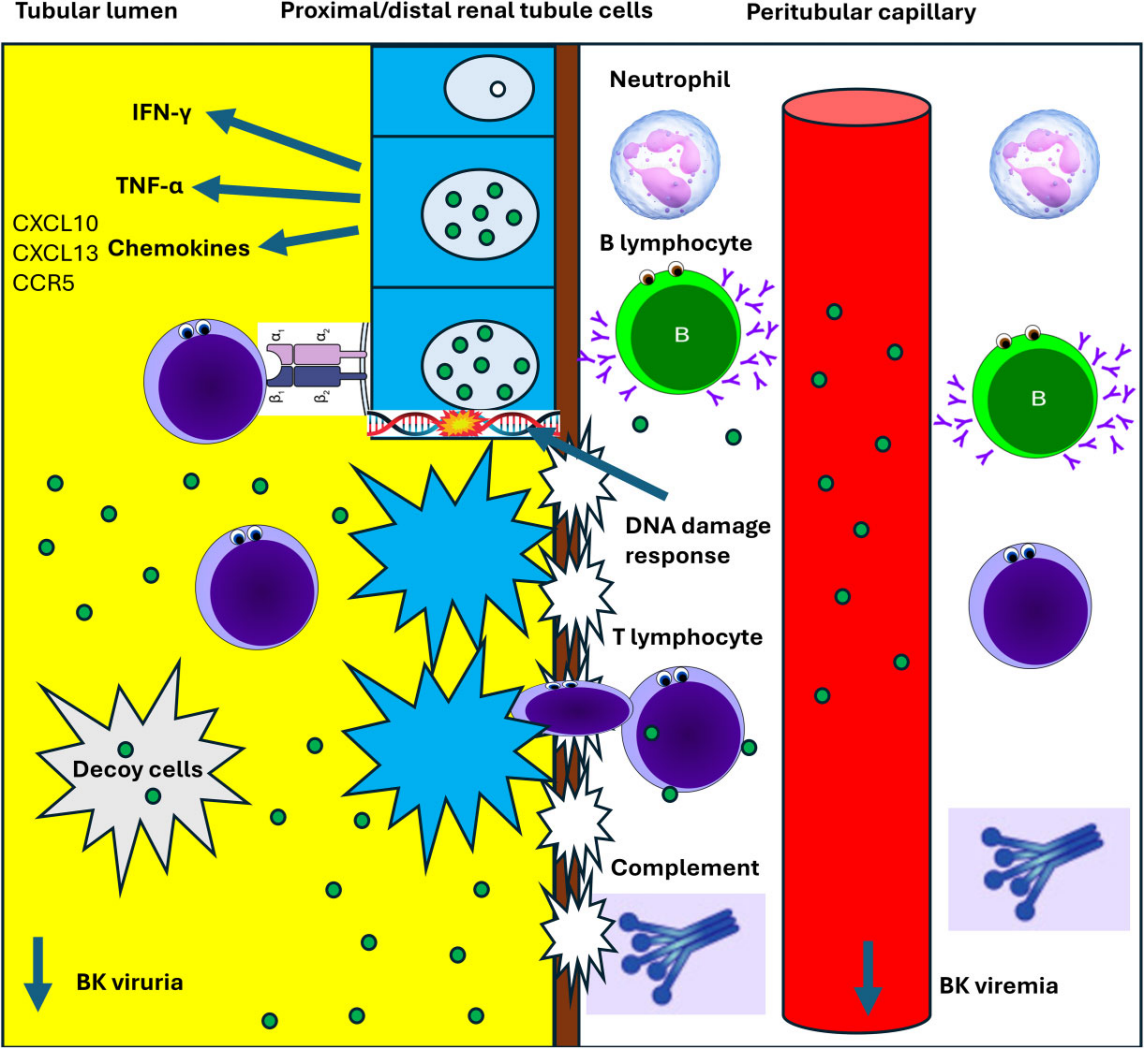
Visualization of BKPyVAN-associated pathways from transcriptomic characterization in the posttransplant kidney tissue.

Host-related gene pathways were extracted from the written text, tables, figures, and supplementary material of seven studies that contained a method of pathway analysis, where the identified pathways and study of origin were reported (Table 4). It should be noted that three studies performed primarily a DEG/transcriptomic signature-related analyses and did not report pathway results within their analyses (Mannon et al. 2005, Sigdel et al. 2016, Adam et al. 2020). In addition to the three studies that did not include pathway analyses, Halloran et al. (2021) specifically highlighted BKPyVAN-associated pathways that were independent of TCMR rejection pathology (excluding several pathways that were similar in both pathologies, such as cytokine, chemokine, and T-cell-related pathways). Overall, there appeared to be a strong agreement among the resulting studies that Th1-related cytokine signalling- (TNFα, IFNγ) (Girmanova et al. 2012, Lubetzky et al. 2014, Jia et al. 2018, Liu et al. 2020, Zhao et al. 2022, Sato et al. 2023), chemokine signalling- (Girmanova et al. 2012, Jia et al. 2018, Liu et al. 2020, Zhao et al. 2022, Sato et al. 2023), and the activation/signalling of T- (Girmanova et al. 2012, Lubetzky et al. 2014, Liu et al. 2020, Sato et al. 2023) and B-lymphocytes (Girmanova et al. 2012, Lubetzky et al. 2014, Halloran et al. 2021) were key pathways that differentiated BKPyVAN and stable graft function (Table 4). Three studies (Halloran et al. 2021, Sato et al. 2023, Yang et al. 2023) agreed upon the upregulation of viral-specific pathways (DNA replication-, DNA strand elongation-, cell division-, cell proliferation-, cell cycle checkpoints-, and DNA damage repair) revealing the ability of BK polyomavirus to manipulate the cell transcriptome. These viral specific pathways are most likely critical to BKPyVAN pathology, however the other studies were primarily focused on characterizing aberrant immune-related pathways. Sato et al. (2023) was the only study to report the significant activation of the PD-1 signalling pathway (T-cell inhibitory pathway), this study was primarily focused on the novel similarities between BKPyVAN and renal cell carcinoma. The activation of PD-1 signalling may be a novel mechanism contributing to BKPyVAN, however it is yet to be agreed upon and requires further evidence. Only one study reported the activation of complement- and neutrophil-related pathways, however there were three studies that observed significantly upregulated DEGs in relation to the complement system. Although innate immunity is likely to play a significant role in detecting BKPyV DNA and controlling infection, our resulting studies did not emphasize the role of innate immunity in their pathway analyses and reporting. Therefore, this further highlights that clarity is required to determine the potential role of innate immune mechanisms (such as neutrophil infiltration and/or complement activation) in BKPyVAN pathology. Altered pathways associated with BKPyVAN posttransplant kidney tissue are visualized in Fig. 2.

**Table 4.** The summary of the top gene pathways associated with BKPyVAN and the studies they were identified in.

BKPyVAN versus Stable, biological pathways reported (*P <* .05)	Citations
Cytokine-related pathways (incorporating NF-kB transcription, IFNγ signalling, TNF cytokine signalling, Interleukin signalling, cytokine-cytokine receptor)	Girmanova et al. (2012), Lubetzky et al. (2014), Jia et al. (2018), Liu et al. (2020), Zhao et al. (2022), Sato et al. (2023)
Chemokine-related pathways	Girmanova et al. (2012), Jia et al. (2018), Liu et al. (2020), Zhao et al. (2022), Sato et al. (2023)
T-cell-associated pathways (incorporating Th2 cell differentiation, T-cell activation, T-cell receptor signaling, quantitative cytotoxic T-cell)	Girmanova et al. (2012), Lubetzky et al. (2014), Liu et al. (2020), Sato et al. (2023)
B-cell-associated pathways (incorporating B-cell proliferation and receptor signalling)	Girmanova et al. (2012), Lubetzky et al. (2014), Halloran et al. (2021)
Calcium-ion signalling and transport	Jia et al. (2018), Zhao et al. (2022)
Viral-associated pathways (incorporating DNA replication-, DNA strand elongation-, cell division-, cell proliferation-, cell cycle checkpoints-, and DNA damage repair)	Halloran et al. (2021), Sato et al. (2023), Yang et al. (2023)
Neutrophil-related pathways	Sato et al. (2023)
Complement-related pathway	Jia et al. (2018)
Inhibitory-associated pathway (incorporating PD1 signalling)	Sato et al. (2023)

### Immune-related transcriptomic profile of BKPyVAN

The comparison of BKPyVAN and stable functioning transplant groups led to the identification of several DEGs and enrichment of pathways associated with severe immune pathology. A common theme was the upregulation of chemokine signalling pathways linked to immune cell trafficking (*CXCL10, CXCL9, CCR7, CCL5*, and *CXCR3*), proinflammatory cytokine activation (*TNFα, IFNγ*, and *interleukins*), T-cell activation (CD28, CD80, and CD86), and cytotoxicity molecules (PRF1, GZMA, GZMB, and GZMK) in BKPyVAN (Mannon et al. 2005, Girmanova et al. 2012, Lubetzky et al. 2014, Sigdel et al. 2016, Liu et al. 2020, Zhao et al. 2022). Other notable pathways that were reported to be enriched in BKPyVAN included complement cascade (Jia et al. 2018), neutrophil degranulation (Sato et al. 2023), calcium-mediated signalling (Jia et al. 2018, Zhao et al. 2022), PD-1 signalling (Sato et al. 2023), and B-cell transcript activity (Girmanova et al. 2012, Lubetzky et al. 2014, Halloran et al. 2021). The extensive cytokine and chemokine expression in the renal biopsies of BKPyVAN patients, likely contributes to the heightened inflammation and may contribute to severe disease outcomes such as allograft dysfunction and failure. In the studies reviewed, the cytokine-related pathways that were elevated in BKPyVAN, included TNFα-, IFNγ-, IL1-, IL2-, IL4-, IL6-, IL8-, IL10-, and IL13-signalling pathways. From the studies we have investigated, there was significant enrichment of both Th1- (TNFα, IFNγ, and IL2) and Th2- (IL4, IL10, and IL13) cell responses. Significant enrichment of Th2 cytokines (IL4-, IL10-, and IL13-signalling pathways), in the context of viral infection, can inhibit protective Th1 antiviral responses leading to severe immune pathology and delayed clearance of virus (Aleebrahim-Dehkordi et al. 2022). An appropriate Th1 dominant immune response is effective in clearing viral infections, however a cytokine storm induced Th2 dominant response has been associated with poor clinical outcomes (Aleebrahim-Dehkordi et al. 2022).

## Discussion

In this scoping review, we explored published literature on the use of transcriptomic technologies to identify molecular biomarkers and novel pathways that play a critical role for BKPyVAN in KTRs. We identified 11 papers that examined transcriptomic data in human kidney tissue and observed the use of multiple transcriptomic technologies, which included whole genome-, digital based-, PCR based-, and single cell microarrays. Several studies reported DEG and molecular pathways altered in BKPyVAN compared with stable functioning grafts. A range of host markers were associated with BKPyVAN at the transcript level, mainly immune-related, as well as BKPyV-associated genes (LTAg, VP1, VP2, Agnoprotein, and VP3). Significant upregulation of pathways observed for BKPyVAN in both innate and adaptive immunities, which included chemokine-, cytokine-, cytotoxicity-, and T-cell signalling. In addition, the ability of BKPyV to manipulate the cell cycle checkpoint-, DNA repair-, and cell proliferation pathways was observed, showing similarities to the transcriptional gene patterns of tumour microenvironments. There is limited evidence regarding the differences between BKPyVAN and other rejection pathologies, and similarities noted between inflammatory pathways involved in BKPyVAN and TCMR that need further exploration. The published studies are also limited by the use of small sample sizes of BKPyVAN with only 3–15 BKPyVAN patient cohorts available in publicly available datasets, with DEG identified remaining unvalidated.

Balancing BKPyVAN and allograft rejection remains a challenging clinical case in transplant centres worldwide. A reduction of immunosuppression is required to restore BKPyV-specific T-cell function and suppress BKPyV activity, however this can paradoxically lead to TCMR with the infiltration of alloantigen-specific T-cells. A key challenge is finding the right balance of immunosuppression resulting in reduction of viral replication without increasing the risk of rejection. In fact, immunosuppression reduction in the setting of BKPyV viremia was linked to the generation of *de novo* donor specific antibodies, supporting the need for close monitoring (Hod-Dvorai et al. 2023). The exact mechanism of correlation between BKPyVAN and TCMR is yet to be fully understood. Exposure to either viral- and/or allo-antigens results in the activation of T-cell-mediated inflammation and tissue injury, which suggests that similar immune pathways (chemokine, cytokine, cytotoxicity, and T-cell signalling) are at play in both BKPyVAN and TCMR. BKPyV infection leads to severe epithelial cell injury, releasing damage associated molecular patterns that activate the innate immune system and the production of chemokines/cytokines. This leads to tissue injury, fibrosis, nephron loss, and activation of the adaptive immune system inducing rejection (Zhao et al. 2018, Eleftheriadis et al. 2021). The significant enrichment of the programmed cell death 1 (PD-1) signalling pathway also suggests the involvement of chronic inflammation and T-cell exhaustion in BKPyVAN, which may be the result of chronic exposure to viral- and/or allo-antigens (Jubel et al. 2020). Therefore, the comparison of BKPyVAN and TCMR cohorts at the transcriptomic level are needed to provide greater evidence on the similarities and differences of the pathological pathways involved.

A common theme throughout the review was the upregulation of chemokine signalling pathways, proinflammatory cytokines, T-cell activation, and cytotoxicity molecules in BKPyVAN pathology. Significant Th2 cell response (identified by the presence of IL4, IL10, and IL13-signalling) may inhibit the protective Th1 response against BKPyV further delaying the clearance of BKPyV viral load and leading to poor prognosis. This is the case in viral infection and cancer where a dominant Th1 response drives antiviral and antitumour mechanisms, while Th2 dominant cell responses may inhibit host antitumor and antiviral immunity leading to poor prognosis and lower survival rates. BKPyVAN pathology was also observed to have a significant infiltration of cytotoxic cells (T-cells and NK cells) highlighted by the expression of genes associated with cell death/apoptosis. In our study, we have also highlighted the significant upregulation of CXCL10 mRNA in BKPyVAN posttransplant kidney tissue in agreement with other studies (Haller et al. 2023). A recent study of urine CXCL10 showed significant upregulation in BKPyV viruria (especially with decoy cell shedding) and there was a high certainty that low urine CXCL10 values could rule out the presence of ≥ 3 decoy cells and BKPyV DNAemia (Haller et al. 2023).

Overactivation of the TNF-signalling pathway seemingly contributes to the pathophysiology of BKPyVAN rather than protecting transplanted kidneys from BKPyV infection (Li et al. 2023). TNFα is amongst the best-characterized inducers of the NF-κB-signalling pathway, which has been identified as a proinflammatory pathway associated with BKPyVAN. TNFα-signalling is largely implicated in many forms of renal inflammatory diseases and is generally not detected in stable functioning allografts. Renal transplant complications such as ischemia-reperfusion injury, acute TCMR and viral infections are known to trigger TNF-signalling pathways (Li et al. 2023). Some viruses have evolved to take advantage of TNF-signalling pathways to enhance replication. It has been reported that cytomegalovirus (CMV) and human simplex virus type 1 are activated by TNF-α, exacerbating viral replication and inflammation (Walev et al. 1995, Wiggins et al. 2000). Hence, the overexpression of TNF-α and its associated pathways may result in amplified BKPyVAN pathology. This suggests that blockade of the TNF-signalling pathway may have a therapeutic benefit in treating severe cases of BKPyV infection. However, a greater clarity about the contribution of TNF-signalling to antiviral and pathological mechanisms in BKPyVAN is required.

Interferon signalling pathways are also a potent line of defence against viral infections. Many studies have demonstrated a strong negative correlation between IFN-γ producing cells and the development of BK viremia (Mueller et al. 2011). However, it was noted in this review that IFN-γ signalling pathways are significantly enriched in BKPyVAN renal biopsies compared to stable functioning KTRs in multiple studies. Therefore, the role of IFN-γ cytokine in BKPyVAN pathology may play a therapeutic effect in decreasing viral load via Th1 infiltrating cells, however excess IFN-γ expression may originate from a nonspecific immune response or a direct immune response against alloantigens. The study also highlighted that the transcriptomic gene patterns of BKPyVAN show great similarity to various cancer pathologies. It is well established that BKPyV alters cell cycle-, DNA damage repair-, and cellular proliferation-signalling pathways for BKPyV DNA replication, which are heavily involved in the occurrence and progression of multiple cancers.

This review has identified several limitations, including the paucity of studies of this nature, the small sample sizes of BKPyVAN patients, technical variations including gene expression platforms and statistical methodologies, lack of validation studies, and limitations regarding study design (all cross-sectional in nature). One of the major limitations of the data published in this area is the use of multiple mRNA platform types. The whole genome-based microarrays were able to evaluate the gene expression of 25 000 mRNA transcripts, however they may not all be relevant in the context of renal transplantation and BKPyVAN pathology. The low-density PCR-based platforms (90 mRNA transcripts) from investigations conducted more than a decade ago were limited by the number of transcripts that underwent evaluation and digital-based Nanostring Pan-cancer microarray (up to 800 mRNA transcripts) employed in the study of Adam et al. (2020) had not been validated for the purpose of rejection-based pathology or infectious complications in transplant, such as BKPyV and CMV (Mengel et al. 2020). There has been a concerted effort from the Banff molecular diagnostic working group to design a diagnostic gene expression panel (Nanostring B-HOT panel) that incorporates validated markers for transplant pathology (Mengel et al. 2020). This will allow the collaboration of transplant centres to develop strongly validated and reliable biomarkers that make a clinical impact, and future studies incorporating this panel will improve these limitations. Ability to measure FFPE tissue with these methodological advances will also provide greater molecular to histological correlation.

Each study provided its own unique parameters and statistical tests to identify DEGs, while also using different platforms to identify significantly enriched pathways. In addition, all studies only showed a cross-sectional view of the transcriptomic gene profile at BKPyVAN diagnosis, therefore, the longitudinal aspects of the immunological response before, during and after BKPyV infection remain lacking. While the longitudinal transcriptomic analysis with noninvasive specimens (urine and serum) can be completed relatively noninvasively, transcriptomic analyses of posttransplant kidney biopsies at multiple time points require ethical consideration due to the invasive nature of the procedure. Thus, there are difficulties in tracking immunological changes with detailed intragraft transcriptomic analyses across shorter durations. Longitudinal study designs are beneficial in understanding how gene expression levels change over time, especially in treatment responsiveness and alterations to treatment plans. The implementation of BKPyVAN longitudinal studies will allow the integration of molecular profiling, clinical characteristics, and histological features before and after intervention treatments, providing transplant nephrologists with the necessary information to guide evidence-based treatment plans. Cross-sectional studies are a great first step in identifying DEG in the kidney microenvironment, however longitudinal studies will be essential in guiding effective treatment plans for BKPyVAN. The lack of cross-sectional studies analysing the transcriptomic profile of BKPyVAN kidney tissue in large KTR cohorts highlights the need for original research. Another limitation is that most of these biomarkers are in the large part exploratory, with a lack of follow up validation. Due to the heterogeneous nature of BKPyVAN pathology, a combination approach of a transcriptomic signature (containing several transcripts) is likely to be more predictive than a single transcript. These transcriptomic signatures may be useful in predicting KTRs at high risk of BKPyVAN, more sensitively detecting BKPyVAN, and/or predicting treatment responsiveness or graft outcome.

The original transcriptomic studies of BKPyVAN kidney biopsies were completed with small cohorts of patients. This is evident in the three publicly available datasets which included between 3 and 15 BKPyVAN patients with GSE72925 (*n* = 10), GSE75693 (*n* = 15), and GSE47199 (*n* = 3). The studies of Adam et al. (2020) (*n* = 60) and Halloran et al. (2021) (*n* = 50) provided significantly larger cohort of BKPyVAN patients for transcriptomic analysis, however these studies did not provide a publicly available transcriptomic dataset for evaluation. It must be noted that three bioinformatic studies (Liu et al. 2020, Zhao et al. 2022, Sato et al. 2023) identified in this review repetitively analysed the same NCBI GEO datasets (GSE72925, GSE75693, and GSE47199), further demonstrating the lack of original transcriptomic research associated with BKPyVAN in KTRs. Therefore, this scoping review emphasizes the need to produce original transcriptomic data with large BKPyVAN cohorts to validate the potential of transcriptomic markers, instead of bioinformatic studies analysing the same datasets to avoid verification bias.

The adoption of high-throughput transcriptomic technologies will simplify large and labour-intensive experiments, leading to rapid evaluations of biological processes and interventions/treatment plans in a clinical environment. However, the use of transcriptomic biomarkers for both risk prediction and therapeutic purposes must be carefully assessed before implementation into the clinical environment. Although recent technologies (whole genome- and digital-microarrays) allow transcriptomic evaluation of the whole renal biopsy, they are not able to measure gene expression within a specific area of interest. Hence, current technologies may not provide a complete representation of the pathology at hand, as many diseases are localized to a specific area or cell type within the tissue. There is potential to determine gene expression profiles within a focused region of pathology or a particular cell, especially with the implementation of single cell transcriptomic and spatial transcriptomic technologies. These technologies are not likely to be used regularly in clinical practice but will be critical in discerning similarities/differences between BKPyVAN and TCMR. As according to our search criteria, there has only been one study (Yang et al. 2023) that has analysed human posttransplant kidney tissue with BKPyVAN via new transcriptomic technologies such as single cell RNA-seq. This study identified novel genes associated with BKPyV infection of renal proximal tubule epithelial cells in human kidney allografts, which is associated with poor outcomes in BKPyVAN (Yang et al. 2023). It was evident that the DEG associated with BKPyV infection of RPTECs were associated with cell proliferation (MKI67 and TMPO) and not with inflammatory/immune related genes as observed in the whole renal biopsy (Yang et al. 2023). This is logical as the inflammation observed in BKPyVAN is not isolated to renal tubule cell epithelium and can be observed in both the tubular structure and interstitial area. We excluded two studies (An et al. 2021, Weissbach et al. 2024) with the application of scRNAseq technology in a BKPyV-infected primary renal proximal tubule epithelial cell (RPTEC) *in vitro* model. Weissbach et al. (2024) revealed a novel association of BKPyV late viral gene expression and a mitochondrial stress pattern (upregulation and downregulation of mitochondrial-encoded and nucleus-encoded mitochondrial genes, respectively). Whereas, An et al. (2021) observed downregulation of drug metabolism and detoxification-, tumour necrosis factor signalling-, and energy metabolism-pathways in RPTECs expressing moderate or high levels of viral mRNAs compared to low viral levels/mock cells. We acknowledge the novelty of using scRNA-seq to characterize BKPyV infection *in vitro*, however there are limitations as BKPyVAN is most likely a combination of both viral- and alloimmune- induced pathology, which is difficult to simulate in these models. Furthermore, given the complexity of allograft rejection it is more accurate to use *in vivo* tissue. Although spatial transcriptomics are likely to unveil important insights into the pathogenesis of BKPyVAN in the future, there were no studies currently that were able to fit this criterion.

This scoping review contained several strengths including the novelty of the topic and the ability to assess the DEG and pathological mechanisms identified in BKPyVAN. The transcriptomic comparison of BKPyVAN and stable functioning KTRs across multiple studies has enabled a better understanding of the pathology occurring in BKPyVAN, whereas the BKPyVAN and TCMR comparisons have highlighted the almost identical transcriptomic profile observed in both pathologies. This raises questions around the pathogenesis of BKPyVAN, especially the trigger (viral- and/or allo-antigens) for T-cell infiltration and its contribution to the severity of pathology. The review also highlights the importance of integrating viral transcripts into the analyses of BKPyVAN pathology, as this will be an important first step in observing the relationship between mRNA expression of viral transcripts and immune/inflammatory mediators. This information will be critical in discriminating if BKPyVAN inflammatory pathology is of viral- or rejection-origin. However, it is critical that DEG, pathological mechanisms, and treatment targets are independently validated in BKPyVAN cohorts with greater sample sizes and that transplant centres are completing assessment of biopsies on a single transcriptomic platform to encourage multicentre collaboration and strongly validated biomarkers.

## Conclusion

This review has demonstrated the potential of gene expression markers and enriched pathways identified from transcriptomic investigations to enhance our understanding of BKPyVAN pathology. There is currently no strong evidence for an antiviral treatment that can treat BKPyV infection or BKPyVAN directly. Therefore, identifying a mechanism of action and novel treatment targets for BKPyVAN through transcriptomics is needed to improve BKPyV infection outcomes in KTRs. The review also highlights the importance of integrating BKPyV viral transcripts into the analyses of BKPyVAN pathology, for further observation of the relationship between mRNA expression of viral transcripts and immune/inflammatory mediators. This information will be critical in discriminating if BKPyVAN inflammatory pathology is of viral- or rejection-origin.

Identifying clinically useful viral and gene expression signatures that can identify BKPyVAN- and TCMR-related pathology to predict treatment responsiveness and clinical outcomes would be the next step towards precision biomarker-guided treatment and management strategies and great improvements in the clinic. This review demonstrates clear knowledge gaps and underutilization of transcriptomic profiling in BKPyVAN, which need to be addressed by well powered multicentre collaborations and longitudinal study designs employing novel technologies in this area. Therefore, transcriptomic interpretation of BKPyVAN in KTRs is required to identify pathological mechanisms and discern similarities and differences with TCMR pathology, identify treatment targets and effectively guide treatment and management strategies.
